# Valsalva Maneuver Versus Carotid Sinus Massage for Supraventricular Tachycardia: A Systematic Review and Meta-Analysis of Randomized Controlled Trials

**DOI:** 10.7759/cureus.70064

**Published:** 2024-09-24

**Authors:** Abdullatif A Alfehaid, Osama T Almutairi, Mohammed H Albloushi, Ahmad A Alahmad, Malek K Hasan, Omar F Alawadhi, Abdullah A Alibrahim, Abdulrahman K Alfailakawi, Mshal Alhatm, Fahad N Almuhannadi, Abdullah W Alshuaib, Abdullah M Alharran

**Affiliations:** 1 College of Medicine, Royal College of Surgeons in Ireland, Busaiteen, BHR; 2 College of Medicine, Royal College of Surgeons in Ireland, Dublin, IRL; 3 College of Medicine, Alfaisal University, Riyadh, SAU; 4 College of Medicine and Medical Sciences, Arabian Gulf University, Manama, BHR

**Keywords:** carotid sinus massage, paroxysmal supraventricular tachycardia, supraventricular tachycardia, svt, vagal maneuvers, valsalva maneuver

## Abstract

Supraventricular tachycardia (SVT) is one of the most common cardiac arrhythmias, characterized by a sudden increase in heart rate. Initial management often involves vagal maneuvers, including the Valsalva maneuver (VM) and carotid sinus massage (CSM). VM can be categorized into standard VM (sVM) and modified VM (mVM). This study aimed to synthesize the first evidence from published randomized controlled trials (RCTs) comparing the efficacy of VM versus CSM. A comprehensive search across databases, including PubMed, Web of Science, Scopus, Cochrane Library, and Google Scholar, was conducted up to July 29, 2024. The primary endpoint was the success rate of converting SVT to sinus rhythm. The dichotomous outcome was analyzed using a fixed-effect model to calculate the risk ratio (RR) and 95% confidence intervals (CI). The Risk of Bias (RoB) tool, version 2, was employed to assess bias in the included RCTs. In total, three RCTs with 346 cases were analyzed. Concerns were noted regarding potential bias related to the randomization process in all three studies. The meta-analysis of these RCTs (comprising four arms) revealed that VM had a higher success rate than CSM for treating SVT, with an RR of 1.82 (95% CI: 1.29-2.57, p<0.001). Subgroup analysis showed that the rate of conversion to sinus rhythm was significantly higher in the sVM compared to CSM (RR=1.61, 95% CI (1.13-2.29), p=0.01). Additionally, subgroup analysis of one study indicated that mVM was associated with a higher rate of SVT conversion to sinus rhythm compared to CSM (RR=9.28, 95% CI (1.25-69.13), p=0.03). In conclusion, VM demonstrated a higher success rate compared to CSM in treating SVT. Specifically, mVM was more effective than CSM in both terminating SVT and restoring sinus rhythm, though this evidence was based on a single RCT; hence, the related conclusion should be interpreted with caution and requires validation using additional RCTs. Further research in diverse patient populations and clinical settings is necessary to validate these findings and potentially support the broader use of mVM in practice. Additional well-designed, multi-center studies with diverse populations are needed to confirm these observations and provide more comprehensive guidance on SVT management. This is important to enhance the generalizability of results across different demographics and clinical settings. This approach helps ensure that treatment effectiveness is applicable to a broader range of patients, accounting for variations in age, gender, comorbidities, and regional practices.

## Introduction and background

Supraventricular tachycardia (SVT) is defined as rapid heartbeats that start above the ventricles (i.e., in the atria or atrioventricular node), and it is a common cardiac arrhythmia [1,2]. It includes several subtypes, such as atrial tachycardia, atrioventricular reciprocating tachycardia, and atrioventricular nodal reentrant tachycardia [3]. Atrial tachycardia is characterized by rapid electrical activity originating from the atria. It can result in palpitations, dizziness, or fatigue. It may occur in patients with underlying heart conditions or as a result of structural abnormalities [3]. Atrioventricular reciprocating tachycardia involves an accessory pathway that allows reentrant circuits between the atria and ventricles. It is often associated with Wolff-Parkinson-White syndrome. Symptoms can include palpitations and chest pain, and it can lead to more serious complications if not treated [3]. Atrioventricular nodal reentrant tachycardia occurs due to a reentrant circuit within or near the atrioventricular node. It is the most common form of SVT and typically presents with rapid heart rates, often with minimal hemodynamic impact. Treatment may be needed for symptomatic relief [3]. Heart rates commonly range from 150 to 220 beats per minute, with quick onset and abrupt termination characteristic of SVT [4]. Although detection and treatment techniques are improving, there is little chance of recurrence and a >90% success rate with catheter ablation, making it an extremely successful treatment [5]. However, catheter ablation is a more invasive procedure and may not be suitable for all patients. Therefore, there is a growing need for more accessible, effective, safe, and convenient bedside methods, such as the Valsalva maneuver (VM) and carotid sinus massage (CSM).

Standard VM (sVM) is a medical procedure that involves forcefully exhaling while keeping the airway closed, resulting in increased pressure within the chest cavity to around 40 mmHg for a duration of 15-20 seconds [6]. It has been extensively utilized in physical examinations and medical treatments [7]. A more efficient and straightforward version of the VM has been created in comparison to the conventional method [8]. Although usually secure, a high level of caution and consideration must be taken in its intraoperative use [6]. A better form of the sVM, the modified VM (mVM), is used to treat SVT. It entails lifting the lower limbs against resistance upon expiration [9]. Elevating both legs right after performing the sVM can enhance blood return to the heart, which raises jugular vein pressure. This increase in pressure boosts vagal tone, stimulates the vagus nerve, and leads to a reduction in heart rate [9]. A recent meta-analysis of 19 randomized controlled trials (RCTs) by Lu and colleagues demonstrated that mVM outperformed sVM in achieving higher cardioversion rates in SVT patients without increasing adverse effects [10]. While pharmacological options (particularly adenosine) and vagal maneuvers (particularly mVM) provide immediate relief, catheter ablation offers a more definitive, long-term solution for SVT, despite being an invasive procedure [11].

CSM is a diagnostic and therapeutic technique particularly effective for managing tachyarrhythmias and addressing hypersensitivity of the carotid sinus [12,13]. There have been concerns raised about the safety and effectiveness of CSM [13]. According to some experts, treating SVT with pharmaceutical therapy and other techniques like the VM may be safer and more successful [14]. The safety of CSM is still a source of worry, particularly in elderly patients who are more likely to have atheromatous carotid arteries, which can increase the risk of subsequent strokes [14]. Furthermore, it has been noted that CSM may increase the risk of transient hypotension accompanied by a strong vagal response, which can lead to significant bradycardia or even asystole in some individuals, especially those with underlying heart conditions [15]. In light of these factors, it might be necessary to reevaluate the safety and efficacy of CSM as a first-line therapy intervention [16].

Recent RCTs [17-19] have compared the effectiveness of VM versus CSM in terms of success rate outcome among patients with SVT. However, the results have never been summarized previously. The aim of this first-ever systematic review and meta-analysis is to synthesize evidence from published RCTs to evaluate the comparative efficacy of VM (standard and modified) and CSM in terminating SVT. We hypothesized that VM would be more effective than CSM in terminating SVT.

## Review

Methods

Protocol Registration

In accordance with the Preferred Reporting Items for Systematic Reviews and Meta-Analyses (PRISMA) guidelines [20] and the Cochrane Handbook for Systematic Reviews of Interventions [21], the research protocol was registered with the International Prospective Register of Systematic Reviews (PROSPERO) with the identification number: CRD42024575512 (https://www.crd.york.ac.uk/prospero/display_record.php?RecordID=575512).

Literature Search Strategy

From the beginning until July 29, 2024, multiple databases were searched for relevant studies, including PubMed, Web of Science, Scopus, Cochrane Library, and Google Scholar. The search study used for this paper included terms such as “Valsalva maneuver," “carotid sinus massage," “supraventricular tachycardia," “paroxysmal supraventricular tachycardia," and “vagal maneuvers." There were no specific filters according to publication year or age of patients. Eligible studies’ citations were also reviewed to include any missed study. Table 1 presents a detailed search strategy used for each database.

**Table 1:** The detailed search strategy used for each database.

Search Strategy	
PubMed	(Supraventricular tachycardia OR Paroxysmal Supraventricular Tachycardia OR SVT) AND (Valsalva Maneuver) AND (Carotid Sinus Massage)
Web of Science	(Supraventricular tachycardia) OR (Paroxysmal Supraventricular Tachycardia) AND (Valsalva Maneuver) AND (Carotid Sinus Massage)
Scopus	("Supraventricular tachycardia" OR "Paroxysmal Supraventricular Tachycardia" OR "SVT") AND ("Valsalva Maneuver") AND ("Carotid Sinus Massage")
Cochrane Library	(Supraventricular tachycardia OR Paroxysmal Supraventricular Tachycardia OR SVT) AND (Valsalva Maneuver) AND (Carotid Sinus Massage)
Google Scholar	("Supraventricular tachycardia" OR "Paroxysmal Supraventricular Tachycardia") AND ("Valsalva Maneuver") AND ("Carotid Sinus Massage")

Inclusion and Exclusion Criteria

The population, intervention, comparator, outcome, and study design (PICOS) inclusion criteria [20] comprised: stable patients presenting with a confirmed diagnosis of SVT based on electrocardiogram (ECG); the intervention group received sVM or mVM; the control group received CSM; the primary endpoint of the success rate of converting SVT into sinus rhythm was reliably reported; and the study design was RCT published in the English language. The exclusion criteria included: non-English publications, abstracts, case reports, conference presentations, editorials, and expert opinions. Additionally, studies were excluded if they involved patients not confirmed to have SVT or non-stable patients with any contraindication to the VM or CSM.

Selection of Studies and Data Extraction

After conducting the initial search for articles, two authors used the Covidence tool to remove duplicates and eliminate studies with titles of low relevance to our research. The remaining studies were then assessed by reading their abstracts. Relevant studies were further evaluated by reviewing their citations to ensure comprehensive coverage. A second pair of authors independently screened the full texts of the selected studies according to the inclusion and exclusion criteria. Any disagreements were resolved by a third author.

Data Collection and Review Outcomes

Data collection from the selected articles was conducted in several stages. First, we extracted citation details, including the study identifier, design, country, setting, duration, and total number of cases. Next, we collected information on the type of VM, whether standard or modified, and the specifics of its administration, such as the timing, pressure, and side of the neck for CSM. We also recorded any rescue interventions and ensured they met the main inclusion criteria for relevance to our study. To clarify, in one study [19], some patients received either the VM or CSM, and if the initial intervention failed, they were given the other option. Each encounter of intervention was treated as a stand-alone case and included in the analysis. The primary outcome for each study was the successful conversion to sinus rhythm in patients. Data were collected independently by two co-authors, and any disagreements were resolved through consultation with the corresponding author.

The Risk of Bias Assessment

The Risk of Bias (RoB) tool version 2 [22] was used to evaluate bias in the included RCTs. Each of the five domains was rated as low risk, high risk, or some concern. The overall quality domain was judged based on the results of the five domains according to the guidelines. Quality assessment was performed independently by two authors, with any disagreements resolved by the corresponding author.

Meta-Analysis

For the pooled analysis, we used Stata (version 18). Dichotomous outcomes were analyzed using a fixed-effect model to calculate the risk ratio (RR) and 95% confidence intervals (CI). Heterogeneity was evaluated using the chi-square test (p<0.1) and the I-squared test (>50%) [23]. Statistical significance for all endpoints was set at a p-value <0.05. Due to the small number of studies included in our analysis (<10), the assessment of publication bias is not considered reliable [24].

Results

Summary of Study Selection

**Figure 1:**
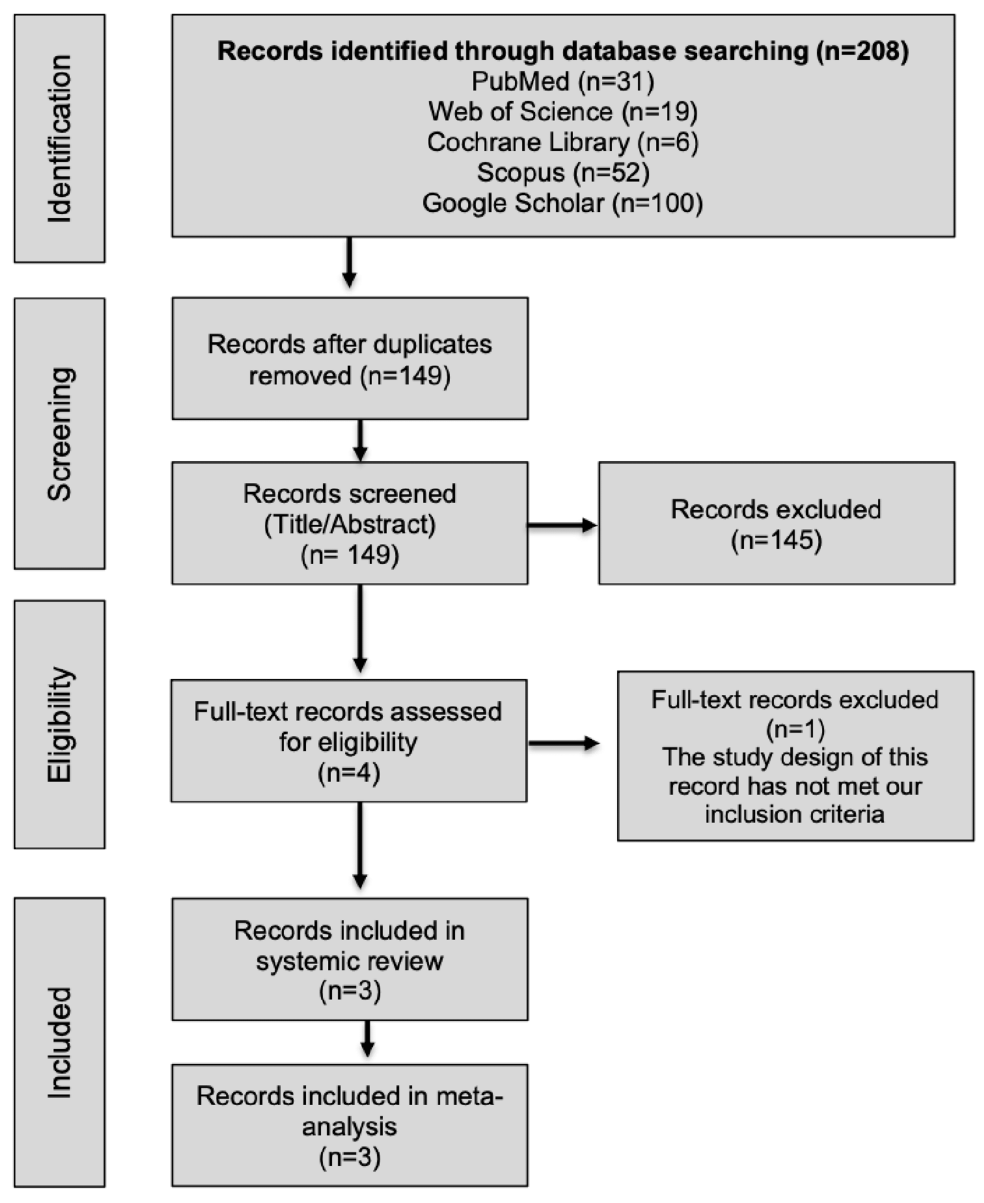
Summary of the Preferred Reporting Items for Systematic Reviews and Meta-Analyses (PRISMA) flowchart and the study selection process.

A total of 208 publications were retrieved from various databases. After removing duplicates, 149 papers were initially screened based on titles and abstracts. Subsequently, four papers underwent full-text screening, of which only three RCTs [17-19] met the inclusion criteria. These three studies were included in both the systematic review and the meta-analysis, as illustrated in the PRISMA flow diagram (Figure 1).

Summary of Included Studies

The three included RCTs were conducted in Turkey, Pakistan, and Singapore, with a total number of 473 cases. All included RCTs were conducted between 1998 and 2019. They were all conducted in an emergency department setting within single centers. Table 2 and Table 3 provide detailed information about the baseline characteristics of the studies and participants included in the research, respectively.

**Table 2:** Summary of the baseline characteristics of the included studies.

Study identifier [reference]	Study design	Country	Total participant's	Setting	Valsalva maneuver type	Duration of the procedure		Rescue intervention	Main inclusion criteria	Primary outcome
						Valsalva maneuver	Carotid sinus massage			
Aslam et al. 2018 [17]	Single-center, randomized controlled trial	Pakistan	100	Emergency department	Standard	40 mmHg for 15 seconds	10-20 seconds per side	Direct current cardioversion or pharmacological treatment	Patients 15-60 years old with supraventricular tachycardia, hemodynamically stable, no contraindications for supraventricular tachycardia	Conversion to sinus rhythm within 10 minutes
Ceylan et al. 2019 [18]	Single-center, randomized controlled trial	Turkey	98	Emergency department	Standard and modified	30-40 mmHg for 20 seconds	10 seconds per side	Adenosine and other antidysrhythmic	Patients >18 years old with supraventricular tachycardia, hemodynamically stable, no contraindications for supraventricular tachycardia	Conversion to sinus rhythm within 5 minutes
Lim et al. 1998 [19]	Single-center, randomized controlled trial	Singapore	275	Emergency department	Standard	40 mm Hg for 30 seconds or more	10 seconds per side	Hemodynamically stable (intravenous calcium channel blocker), hemodynamically unstable (synchronized electrical cardioversion)	Patients >10 years old with supraventricular tachycardia, no contraindications for supraventricular tachycardia	Conversion to sinus rhythm within 10 minutes

**Table 3:** Summary of the baseline characteristics of the included participants. Cases and gender are presented as numbers. Age is presented as means or means ± standard deviations. Vital signs are presented as means (ranges: minimum-maximum).

Study identifier [Reference]	Group	Cases	Age (years)	Gender (male/female)	Vital signs		
					Systolic blood pressure (mmHg)	Diastolic blood pressure (mmHg)	Heartbeat (beats/min)
Aslam et al. 2018 [17]	Standard Valsalva maneuver	50	40.1 ± 13.1	15/35	Not Reported	Not Reported	Not Reported
	Carotid sinus massage	50	38.6 ± 11.8	20/30	Not Reported	Not Reported	Not Reported
Ceylan et al. 2019 [18]	Standard Valsalva maneuver	33	61	14/19	126 (113–138)	76 (71–89)	167 (147–187)
	Modified Valsalva maneuver	32	50	17/15	116 (106–136)	81 (73–90)	177 (165–192)
	Carotid sinus massage	33	63	14/19	118 (103–129)	76 (66–86)	168 (147–183)
Lim et al. 1998 [19]	Standard Valsalva maneuver	139	Not Reported	Not Reported	Not Reported	Not Reported	Not Reported
	Carotid sinus massage	136	Not Reported	Not Reported	Not Reported	Not Reported	Not Reported

Summary of Risk of Bias Assessment

**Figure 2:**
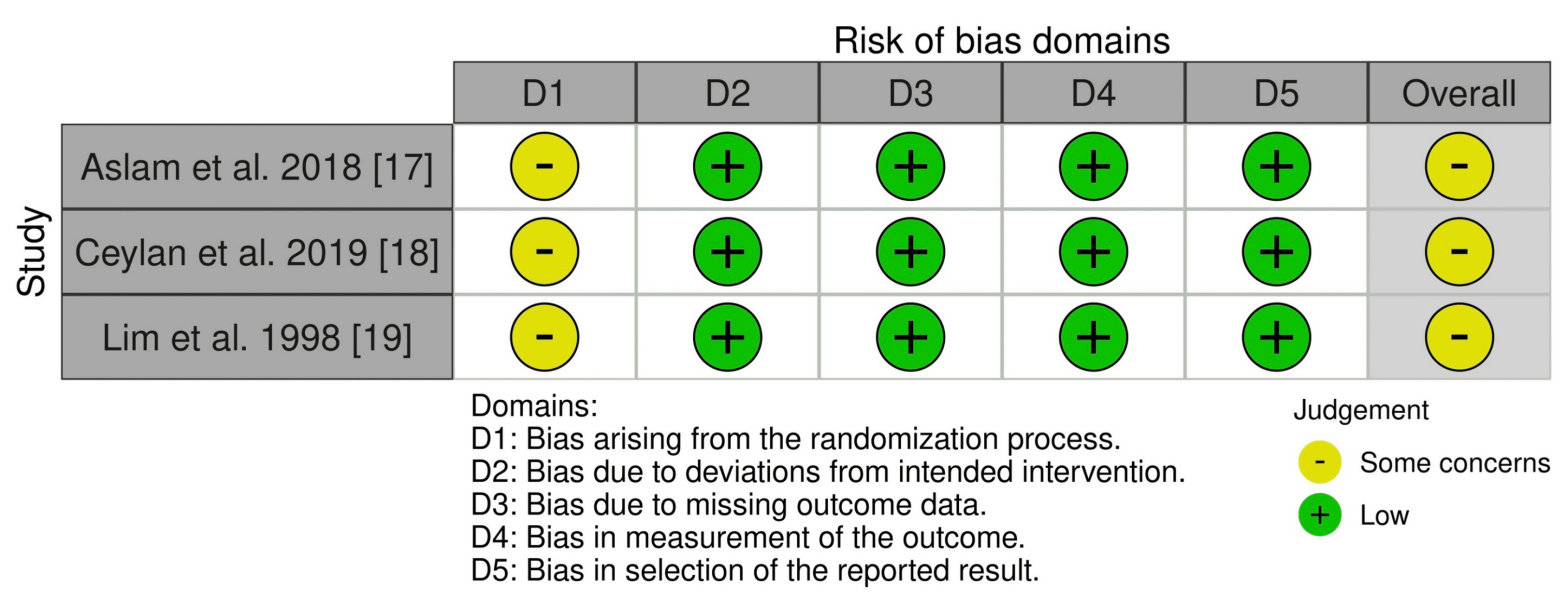
Summary of the risk of bias of the included studies. [17-19]

Figure 2 summarizes the risk of bias assessment for the included RCTs. Overall, there were some concerns about potential bias related to the randomization process. In the study by Aslam et al. 2018 [17], there was insufficient information regarding allocation concealment, introducing potential selection bias. The study by Ceylan et al. 2019 [18] reported unequal distribution of diabetic and hypertensive participants between groups, introducing potential selection bias and variability, impacting the overall interpretation of treatment effectiveness in restoring normal sinus rhythm. In the study by Lim et al. 1998 [19], several key baseline characteristics, including age, gender, and vital signs, were not reported per group, introducing potentially misleading conclusions about the efficacy of the interventions.

Meta-Analysis of the Primary Endpoint

**Figure 3:**
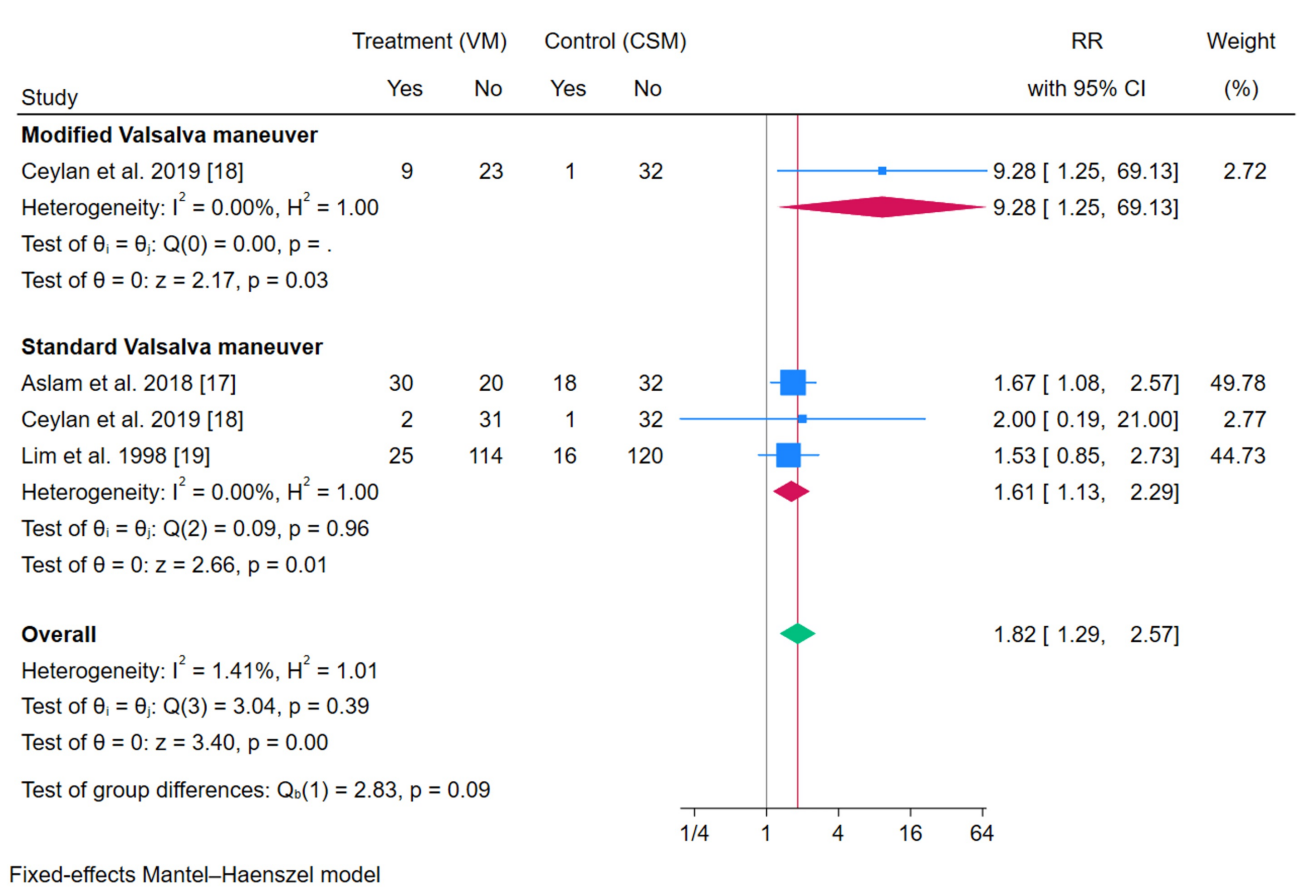
Meta-analysis of the conversion rate of supraventricular tachycardia to sinus rhythm. CI: confidence interval; CSM: carotid sinus massage; VM: Valsalva maneuver [17-19]

Meta-analysis of three RCTs with four arms showed that VM had a higher success rate compared to CSM for treating SVT, with an RR of 1.82 (95% CI: 1.29-2.57, p<0.001). This RR of 1.82 suggests that patients undergoing the VM are 82% more likely to successfully convert SVT to normal sinus rhythm compared to those receiving CSM. The 95% CI of 1.29 to 2.57 indicates that this finding is statistically significant (p<0.001), meaning there is a strong likelihood that the observed effect is not due to random chance. There was no evidence of heterogeneity among the studies (I²=1.41%, p=0.39). Subgroup analysis showed that the rate of conversion to sinus rhythm was significantly higher in the sVM compared to CSM (RR=1.61, 95% CI (1.13-2.29), p=0.01). Additionally, subgroup analysis of one study indicated that mVM was associated with a higher rate of SVT conversion to sinus rhythm compared to CSM (RR=9.28, 95% CI (1.25-69.13), p=0.03) (Figure 3). Leave-one-out sensitivity analysis of the sVM versus CSM showed instability as the omission of the Aslam et al. 2018 study [18] rendered the pooled effect size statistically insignificant (RR=1.56, 95% CI (0.89, 2.74), p=0.125).

Discussion

This meta-analysis of three RCTs comprising 473 cases aimed to compare the efficacy of VM and CSM in caring for SVT. VM had a greater success rate compared to CSM for managing SVT. In particular, mVM proved more effective than CSM in terminating SVT and restoring sinus rhythm, though this conclusion was based on a single RCT; hence, the related conclusion should be interpreted with caution and requires validation using additional RCTs. There was no significant difference between sVM and CSM regarding the conversion to sinus rhythm, although sVM showed a slight trend of being more favorable. The absence of heterogeneity among the included studies [17-19] suggests consistency in the observed effects. 

Numerous studies have compared the effectiveness of mVM over sVM in terminating SVT [25,26]. These studies consistently demonstrate that mVM is significantly more effective than sVM in achieving this goal. The addition of passive leg-raising in mVM is believed to increase venous return to the heart, further activating the baroreceptor reflex and enhancing the parasympathetic response, leading to a higher likelihood of SVT termination [27,28]. Appelboam et al. conducted an RCT in England involving 428 patients with SVT, dividing them equally into two groups: sVM (n=214) and mVM (n=214) [29]. The primary outcome was the return to sinus rhythm at one-minute post-intervention, assessed by the treating clinician and confirmed by an independent investigator. Results showed that 37 (17%) of the sVM group achieved sinus rhythm, compared to 93 (43%) in the mVM group (adjusted odds ratio 3.7, 95% CI 2.3-5.8; p<0.0001). No serious adverse events were reported. The study concluded that the mVM with leg elevation and supine positioning should be considered a routine first-line treatment for patients with SVT and can be easily taught to them [29]. It is important to note that the safety profile of both VM and CSM should be carefully considered. While generally well-tolerated, CSM carries a risk of bradycardia and hypotension, necessitating careful patient selection and monitoring [13,14].

Being the first of its type, this meta-analysis compares the efficacy of CSM, mVM, and SVM in the treatment of SVT. Since the analysis uses only high-quality randomized controlled trials to extract insights, it synthesizes solid data and evidence. Notably, a powerful block randomization strategy was employed in the Ceylan et al. [18] investigation, which reduces the possibility of allocation bias and strengthens the validity of the results.

However, we do need to acknowledge some limitations. The limited number of RCTs and their small sample sizes represent a significant limitation, hindering the power of the study. Two of the included studies [17,19] lacked complete information about the clinical features or vital signs of the patients. Missing such data can significantly affect the interpretation of the success rate for restoring SVT to sinus rhythm. This is because age can influence the physiological response to treatments, as older patients may have different underlying conditions or comorbidities that affect treatment outcomes, potentially skewing success rates if not accounted for. Moreover, gender differences in cardiovascular physiology and responses to interventions can also impact outcomes. If one gender is underrepresented, the overall success rate may not accurately reflect the effectiveness of the treatment across all demographics. Besides, baseline vital signs, such as heart rate and blood pressure, are crucial for assessing the severity of SVT and the patient's overall health. Missing this data can lead to a misinterpretation of how well the intervention worked, as patients with more stable vital signs may respond differently than those with significant instability. 

Since mVM appeared to yield better results, it may be wise to replicate these findings in larger and more diverse populations. Further investigations might consider the underlying mechanisms that account for why mVM demonstrated superiority to sVM. An incomplete understanding of patient compliance, the duration at which strains are applied to lungs for maximal benefit or worse effects, and the value of intrathoracic pressures achieved may help in devising measures to ameliorate further application. Comparative effectiveness studies of vagal maneuvers, if performed in an emergency scenario to treat acute SVT, should help declare the optimal sequencing and treatment strategies. These studies could then help in forming objective guidelines for healthcare centers and improving patient outcomes.

## Conclusions

VM demonstrated a higher success rate compared to CSM in treating SVT. In particular, mVM proved more effective than CSM in terminating SVT and restoring sinus rhythm, though this conclusion was based on a single RCT; hence the related conclusion should be interpreted with caution and requires validation using additional RCTs. It is suggested that mVM could be recommended as a first-line non-pharmacological therapy for SVT patients. However, further research in diverse patient populations and clinical settings is needed to validate these findings and potentially support the broader use of mVM in practice. Additional well-designed, multi-center studies with diverse populations are required to confirm these observations and provide more comprehensive guidance on SVT management. Future trials should focus on head-to-head comparisons of the sVM, mVM, and CSM to establish their relative effectiveness. Additionally, incorporating other non-pharmacological techniques could provide a comprehensive understanding of optimal management strategies for SVT.

## References

[1] Sung RJ, Castellanos A (1980). Supraventricular tachycardia: mechanisms and treatment. Cardiovasc Clin.

[2] Helton MR (2015). Diagnosis and management of common types of supraventricular tachycardia. Am Fam Physician.

[3] Wood K (1995). Mechanisms and clinical manifestations of supraventricular tachycardias. Prog Cardiovasc Nurs.

[4] Wu D (1983). Supraventricular tachycardias. JAMA.

[5] Lindsay BD, Eichling JO, Ambos HD, Cain ME (1992). Radiation exposure to patients and medical personnel during radiofrequency catheter ablation for supraventricular tachycardia. Am J Cardiol.

[6] Kumar CM, Van Zundert AA (2018). Intraoperative Valsalva maneuver: a narrative review. Can J Anaesth.

[7] Nishimura RA, Tajik AJ (2004). The Valsalva maneuver-3 centuries later. Mayo Clin Proc.

[8] Kobat MA, Karasu M (2020). Valsalva and modified valsalva maneuver. J Clin Med Kaz.

[9] Lodewyckx E, Bergs J (2021). Effectiveness of the modified Valsalva manoeuvre in adults with supraventricular tachycardia: a systematic review and meta-analysis. Eur J Emerg Med.

[10] Lu Z, Zhu J, Gao M (2024). Efficacy and safety of modified Valsalva maneuver for treatment of paroxysmal supraventricular tachycardia: a meta-analysis. J Int Med Res.

[11] Chen C, Tam TK, Sun S (2020). A multicenter randomized controlled trial of a modified Valsalva maneuver for cardioversion of supraventricular tachycardias. Am J Emerg Med.

[12] Schweitzer P, Teichholz LE (1985). Carotid sinus massage. Its diagnostic and therapeutic value in arrhythmias. Am J Med.

[13] Pasquier M, Clair M, Pruvot E, Hugli O, Carron PN (2017). Carotid sinus massage. N Engl J Med.

[14] Walsh T, Clinch D, Costelloe A (2006). Carotid sinus massage--how safe is it?. Age Ageing.

[15] Hartig F, Köhler A, Stühlinger M (2018). Carotid sinus syndrome: a case report of an unusual presentation of cardiac arrest while diving. Eur Heart J Case Rep.

[16] Collins NA, Higgins GL 3rd (2015). Reconsidering the effectiveness and safety of carotid sinus massage as a therapeutic intervention in patients with supraventricular tachycardia. Am J Emerg Med.

[17] Aslam M, Raza H, Luqman Luqman, Attar-Rasool S, Luqman S (2018). Comparison of treatment of paroxysmal supraventricular tachycardia by Valsalva manoeuver and carotid sinus massage. P J M H S.

[18] Ceylan E, Ozpolat C, Onur O, Akoglu H, Denizbasi A (2019). Initial and sustained response effects of 3 vagal maneuvers in supraventricular tachycardia: a randomized, clinical trial. J Emerg Med.

[19] Lim SH, Anantharaman V, Teo WS, Goh PP, Tan AT (1998). Comparison of treatment of supraventricular tachycardia by Valsalva maneuver and carotid sinus massage. Ann Emerg Med.

[20] Page MJ, McKenzie JE, Bossuyt PM (2021). The PRISMA 2020 statement: an updated guideline for reporting systematic reviews. BMJ.

[21] Higgins JPT, Thomas J, Chandler J, Cumpston M, Li T, Page MJ, Welch VA (2023). Cochrane Handbook for Systematic Reviews of Interventions. 2nd Edition.. http://www.training.cochrane.org/handbook.

[22] Sterne JA, Savović J, Page MJ (2019). RoB 2: a revised tool for assessing risk of bias in randomised trials. BMJ.

[23] Higgins JP, Thompson SG, Deeks JJ, Altman DG (2003). Measuring inconsistency in meta-analyses. BMJ.

[24] Egger M, Davey Smith G, Schneider M, Minder C (1997). Bias in meta-analysis detected by a simple, graphical test. BMJ.

[25] Abdulhamid AS, Almehmadi F, Ghaddaf AA, Alomari MS, Zagzoog A, Al-Qubbany A (2021). Modified Valsalva versus standard Valsalva for cardioversion of supraventricular tachycardia: systematic review and meta-analysis. Int J Arrhythm.

[26] Lan Q, Han B, Wu F, Peng Y, Zhang Z (2021). Modified Valsalva maneuver for treatment of supraventricular tachycardias: a meta-analysis. Am J Emerg Med.

[27] Walker S, Cutting P (2010). Impact of a modified Valsalva manoeuvre in the termination of paroxysmal supraventricular tachycardia. Emerg Med J.

[28] Smith GD (2016). A modified Valsalva manoeuvre results in greater termination of supraventricular tachycardia than standard Valsalva manoeuvre. Evid Based Med.

[29] Appelboam A, Reuben A, Mann C (2015). Postural modification to the standard Valsalva manoeuvre for emergency treatment of supraventricular tachycardias (REVERT): a randomised controlled trial. Lancet.

